# Traditional Chinese medicine for irritable bowel syndrome: A protocol for meta-analysis

**DOI:** 10.1097/MD.0000000000023394

**Published:** 2020-11-25

**Authors:** Cheng-Jiao Yao, Yi-Lin Li, Meng-Jun Pu, Li-Hong Luo, Pei-Min Feng

**Affiliations:** a Hospital of Chengdu University of Traditional Chinese Medicine, Chengdu; b Department of Geriatrics of the Affiliated Hospital, North Sichuan Medical College, Nanchong; c North Sichuan Medical College, Nanchong, Sichuan, China.

**Keywords:** irritable bowel syndrome, meta-analysis, protocol, traditional Chinese medicine

## Abstract

**Background:**

Irritable bowel syndrom (IBS) is a common functional gastrointestinal disorder which is characterized as recurrent abdominal pain, abdominal discomfort, and abnormal bowel habits such as diarrhea, constipation, both or alternate appear. Although IBS is not fatal, it seriously affects the patients’ daily life and work. Western drug, such as antidiarrheals, gastrointestinal antispasmodic, often cannot get satisfying curative effects. However, the therapeutic effect of Traditional Chinese medicine (TCM) on IBS is very satisfactory which was shown in a large number of randomized controlled trials. Although TCM has been widely used in clinical practice, its relative effectiveness and safety have not been confirmed. Therefore, this study will use meta-analysis to verify the efficacy and safety of different types of TCM in the treatment of IBS.

**Methods:**

We search the China National Knowledge Infrastructure, Wanfang Database, Chinese Science and Technology Periodical Database, Chinese Biomedical Literature Database, Pubmed, Embase, Web of Science, and the Cochrane library for all randomized controlled trial of TCM for the treatment of IBS from their inception to Oct 15, 2020. Two authors will independently select studies, extract data based on predesigned inclusion and exclusion criteria. Methodological quality assessment and risk of bias will be assessed using Cochrane bias risk tool. All data analysis will be conducted using Revman5.3, WinBUGS 1.4.3, and Stata14.2 software.

**Results:**

This study will compare the different outcome indicators of various studies directly and indirectly, and provide a high-quality synthesis of effectiveness and safety of different TCM methods for patients with IBS. The main outcome indicators include effectiveness, remission rate (no drug symptoms), relapse rate, clinical absolute score, and relative score. Secondary outcome indicators included related adverse reactions and serum serotonin concentration.

**Conclusion:**

The conclusion of this systematic review will provide a high-quality evidence based on the efficacy and safety of different TCM treatment methods for IBS.

**Registration number:**

This study protocol has been funded through a protocol registry. The registry number is INPLASY2020100052

## 1 Introduction

Irritable bowel syndrome (IBS) is a common functional gastrointestinal disorder which is characterized as recurrent abdominal pain, abdominal discomfort, and abnormal bowel habits such as diarrhea, constipation, both or alternant appear.^[1]^ The pathogenesis of IBS is complex and it is not yet clear. Studies have found that intestinal motility, visceral hypersensitivity, abnormal brain gut axis regulation, mental and psychological disorders, intestinal immune activation were related to its pathogenesis.^[2]^ IBS is commonly classified into 4 main subtypes: diarrhea predominant (IBS-D), constipation predominant (IBS-C), mixed (IBS-M), and unclassifified (IBS-U), according to its clinical manifestations.^[3]^ Epidemiologic studies have reported a high prevalence of IBS ranging from 5% to 22% in various countries,^[4]^ 2.9% to 15.6% in Asian countries,^[5]^ and 0.82% to 11.5% in China.^[6]^ The incidence rate of IBS around the world increased rapidly with economic growth. Currently, western medicine treatment of IBS include antidiarrheal agents, antispasmodic agents, intestinal prokinetic drugs, visceral analgesics, and so on.^[7]^ These drugs can temporarily relieve abdominal symptoms, but their side effects should not been ignored, let alone relapse. For example, the antidiarrheal agent loperamide can inhibit intestinal peristalsis, prolong the passage time of intestinal contents, and promote the absorption of water, electrolyte, and glucose.^[8]^ However, it can also cause pseudomembranous enteritis, toxic enteritis. Fatal risk of toxic megacolon in patients with ulcerative colitis after taking loperamide. Therefore, we need to further explore new treatment methods for IBS, with good efficacy and small side effects.

Traditional Chinese medicine (TCM) is one of the world's overall medical systems, and has been widely recognized around the world in the long-term medical practice. Traditional Chinese medicine advocates “unity of man and nature.” That means, when treating diseases, we should explore the root causes of macro and micro imbalance of the human body from a holistic view, and restore the balance of the human body through various TCM. This holistic view of TCM is just in line with the characteristics of the diversity of pathogenesis of IBS, which is considered to be promising treatment to improve the symptoms of IBS. In TCM, IBS is classified as “abdominal pain,” “Xiexie” and “Yuzheng.” The etiology of IBS is summarized as abnormal of emotion, diet, and external evil. Liver depression and spleen deficiency, spleen and kidney yang deficiency, cold, and heat are regarded as the key factors of IBS. So there are many corresponding treatment methods for different causes, such as acupuncture, Chinese herbal medicine, and so on. Acupuncture is based on the theory of meridians, which stimulates characteristic acupuncture points to clear meridians and smooth the body's qi and blood.^[9,10]^ Studies have shown that acupuncture can regulate the secretion of serotonin, and then affect the smooth muscle movement of digestive tract.^[11]^ The classic prescription “Tongxie Yaofang” can also play a therapeutic role by affecting the metabolism of tryptophan, methionine, and cysteine.^[12]^ At present, TCM has been widely used for the treatment of IBS due to its characteristics of low price, convenience, high efficacy, and few adverse reactions. Now, there are a series of traditional meta-analysis evaluating the effectiveness between TCM and Western medicine for treating IBS.^[13–16]^ A large part of these papers believed that TCM achieved a better therapeutic effect compared with Western medicine. There are many kinds of TCM treatment methods, different treatment methods have different advantages. However, there is little information that directly or indirectly compares the effectiveness and safety of different TCM treatment methods. Therefore, to solve the above problems, we will use meta-analysis to systematically compare the effectiveness and safety of different TCM interventions, paving the way for future solutions to IBS.

## 2 Methods

### 2.1 Protocol registration

This study protocol has been funded through a protocol registry on the INPLASY website (registration number is INPLASY2020100052). We will strictly abide by the requirements of the “the Preferred Reporting Items for Systematic Review and Meta-analysis Protocols” to report the meta-analysis.^[17]^ If there is any information adjustment during the entire study period, we will promptly correct and update it in the final report.

### 2.2 Inclusion and exclusion criteria

#### 2.2.1 Type of study

Only the study of randomized controlled trial (RCT) can be included, the language will be limited to Chinese or English. Exclude non-RCT, animal experiments, unclear results indicators such as images and other nonquantitative indicators. For the articles published repeatedly in Chinese and English journals, take the latest one published.

#### 2.2.2 Participants

Patients diagnosed with IBS by Rome I, II, III, and IV criteria, not restricted in age, gender, ethnicity, race, and disease stage. Reluctant to accept TCM treatment, patients with severe cardiovascular diseases, mental illnesses, Pregnant women, breast stage, and cancer will be excluded.

#### 2.2.3 Interventions

##### 2.2.3.1 Experimental interventions

The intervention measures of the experimental group were only TCM, such as Chinese herbal medicine, Chinese patent medicine, acupuncture, moxibustion, massage, and so on. It can be monotherapy or combination. RCT comparing the above 2 therapies can also be included, and those who combine Western medicine will be excluded.

##### 2.2.3.2 Control interventions

The control group received conventional treatment of Western medicine, including the use of antidiarrheal agents, antispasmodic agents, intestinal prokinetic drugs, and visceral analgesics.

### 2.3 Outcome indicators

The main outcome indicators include effectiveness (recognized clinical efficacy evaluation criteria), effective including basic recovery, marked effect, improvement; remission rate (no drug symptoms), relapse rate, clinical absolute score, and relative score. Secondary outcome indicators: including any related adverse reactions, the concentration of serum serotonin concentration.

### 2.4 Data sources and search strategies

We will search the following databases: the China National Knowledge Infrastructure, Wanfang Database, Chinese Science and Technology Periodical Database, Chinese Biomedical Literature Database, Pubmed, Embase, Web of Science, and the Cochrane library. Collect all the RCT on the treatment of IBS with TCM. And manually search for references in related literature. The retrieval time is from the inception of the database to Oct 15, 2020. The language is limited to Chinese and English. The search strategy is to combine search terms with subject words and free words. The primary selection process is shown in PubMed search strategy (Table 1).

**Table 1:** Search strategy used in PubMed database.

No	Search items
1	Irritable bowel syndrom
2	abdominal pain
3	Xiexie
4	Yuzheng
5	1 or 2- 4
6	Chinese herbal
7	Traditional Chinese medicine
8	Chinese medicine
9	Chinese traditional medicine
10	TCM
11	Chinese medicine decoction
12	Decoction
13	Herb
14	Acupuncture
15	Needle knife
16	Needle scalpel
17	Acupotomology
18	Fire needling
19	Electroacupuncture
20	Pharmacoacupuncture
21	Massage
22	Tuina
23	Moxibustion
24	6 or 7–23
25	Randomized Controlled Trials
26	Randomized Controlled Trial
27	RCT
28	Control clinical trial
29	Trials
30	25 or 26–29
31	5 and 24 and 30

### 2.5 Selection of studies

**Figure 1:**
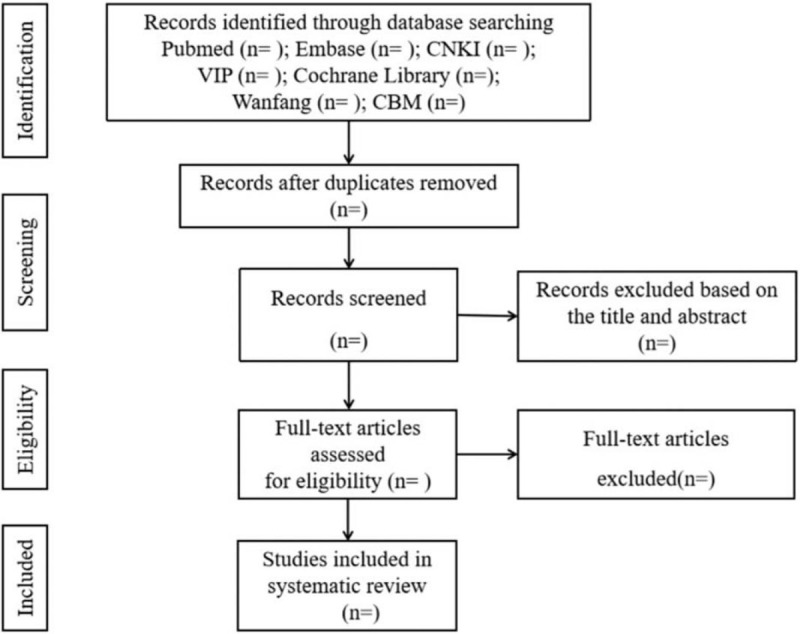
Flow diagram of study selection process.

Two authors independently complete the following process: according to the above search strategy to complete the process of document retrieval, import documents into EndNote X7 for centralized management. Then, according to the inclusion and exclusion criteria, filter the literature by reading the title and abstract. If it is not possible to determine whether the article meets the requirements based on the inclusion and exclusion criteria, then read the full text to select. In the entire literature screening process, if the 2 authors have different opinions, the third author joins the discussion to get a common opinion. The process of research selection is shown in Figure 1.

### 2.6 Data extraction

After the literature search process was completed, the 2 authors independently extracted the following information from the selected study: author, article title, year of publication, contact information, country/region, sample size, participants, diagnostic criteria, baseline characteristics, study design, random methods, blind methods, results, adverse events, and so on, and fill the extracted information into a pre-built Excel table. If necessary, we will contact the trial author for further information.

### 2.7 Dealing with missing data

If there is data loss in the included study, we will contact the original author of the article to obtain the original information. If the missing data is still not available, the existing data will be analyzed and a sensitivity analysis will be performed to address the potential impact of the missing data.

### 2.8 Risk of bias assessment and quality of selected studies

The 2 authors will independently assess the risk of bias (methodological quality) of the included studies based on the bias risk assessment tool recommended in the Cochrane “Risk of bias” assessment tool.^[18]^ Including 7 items: random sequence generation, allocation concealment, blind participants and personnel, blind assessment of results, incomplete result data, selective reports, and other biases. The results in each field will be divided into 3 levels: low bias risk, high bias risk, and unclear bias risk. The 2 authors will exchange assessment results and check whether the assessment results are consistent. If there is a disagreement, the third author will participate in the discussion and determine the final result.

### 2.9 Statistical analysis

Pairwise meta-analyses are conducted by RevMan5.3,^[19]^ Categorical data will be calculated with the risk ratio and 95% confidence intervals (CIs), continuous variables will be reported as mean differences or standardized mean differences with 95% CI. Heterogeneity will be evaluated by Chi-squared test and Higgins *I*^2^ test; If there is no obvious heterogeneity (*I*^2^ ≤ 50% and *P* > .10), the fixed-effect model will be used; otherwise, the random effect model will be applied. Use WinBUGS 1.4.3 and Stata14.2 for network meta-analysis. In WinBUGS 1.4.3 software, Bayesian framework is implemented by the Markov chain Monte Carlo method,^[20]^ which is simulated by 4 chains, the number of iterations is set to 50,000, and the step size is set to 10. At the same time, the Potential Scale Reduced Factor is used to evaluate the convergence of the results. When the PRSF value is approximately equal to 1.00, it indicates that the results are well converged, and the obtained results are highly reliable. If the PRSF is not within this interval, then continue to manually increase the number of iterations 50,000 times until the FRSF is within this range. In the case of many interventions involved, in the evidence network of each outcome indicator, the closed loop formed by the research with direct and indirect evidence needs to be tested for inconsistency. Calculate the inconsistency factor (IF), and judge whether there is inconsistency by the IF value and the P-value. If the IF is close to 0, the 95% CI starts at 0, and *P* > .05, it is considered that the direct comparison and the indirect comparison are consistent.^[21]^ At the same time, the node-split model is used to determine whether each node has local inconsistency. If *P* > .05, there is no obvious inconsistency. If there is no obvious inconsistency between the 2, the consistency model is adopted, otherwise, the inconsistency model is adopted. For the results obtained by the analysis of the consistency model, the stability of the results can be checked by the inconsistency model.^[22,23]^ Make evidence network diagram, correct-compare funnel diagram, and conduct inconsistency test in Stata14.2 software. Simultaneously calculate the value of surface under the cumulative ranking curves and the area under the surface under the cumulative ranking curves curve to rank the efficacy of various interventions. The value range is 0 to 100. The larger the value, the larger the area under the curve indicates the intervention and the greater the likelihood of being the best intervention.

### 2.10 Subgroup analysis and sensitivity analysis

If the Chi-squared test and Higgins *I*^*2*^ test detect obvious heterogeneity between studies, we will conduct a subgroup analysis from the following aspects: different types of TCM, treatment time, IBS classification, stage of disease, and so on. To ensure the Credibility of the research results, we will conduct a sensitivity analysis of the included literature and will eliminate low-quality literature.

### 2.11 Publication bias

If the included studies are sufficient (n ≥ 10),^[24]^ the funnel plot will be used to assess the publication bias of the literature. If the funnel chart has poor symmetry, it indicates publication bias.

### 2.12 Assess the quality of evidence

The evaluation of the strength of the evidence will be based on the grading of recommendations assessment, development, and evaluation system, there are 4 levels of evidence strength: high, medium, low, or very low.

## 3 Discussion

In recent years, a large number of TCM with good curative effect in the treatment of IBS have emerged, which indicates that TCM may served as a promising method to treat IBS in practical application. Yan indicates that acupuncture combined with Chinese herbal medicine is an effective and safe treatment approach for IBS-D patients compared with western medicine.^[25]^ Manheiner has confirmed the effectiveness of acupuncture in relieving the symptoms of IBS.^[26]^ Chinese patent medicine Shugan Jianpi Zhixie has also been proven to be an effective and safe therapeutic option for patients with IBS-D.^[27]^ However, no reports of comparisons between different TCM interventions on IBS has been found. Therefore, this is the first meta-analysis to directly or indirectly compare differences of TCM in treating IBS. It will find out which TCM interventions have best efficacy and safety, and provide the best evidence for clinical practice.

## Author contributions

**Conceptualization:** Cheng-jiao Yao

**Methodology:** Yi-lin Li.

**Project administration:** Meng-jun Pu

**Software:** Li-hong Luo

**Supervision:** Pei-min Feng

**Writing – original draft:** Cheng-jiao Yao

**Writing – review & editing:** Cheng-jiao Yao, Pei-min Feng

## References

[1] Ferreira AI Garrido M Castro-Pocas F Irritable bowel syndrome: news from an old disorder. GE Port J Gastroenterol 2020;27:255–68. 32775547 10.1159/000503757 PMC7383263

[2] Raskov H Burcharth J Pommergaard HC Irritable bowel syndrome, the microbiota and the gut-brain axis. Gut Microbes 2016;7:365–83. 27472486 10.1080/19490976.2016.1218585 PMC5046167

[3] Camilleri M Lasch K Zhou W Irritable bowel syndrome: methods, mechanisms, and pathophysiology. The confluence of increased permeability, inflammation, and pain in irritable bowel syndrome. Am J Physiol Gastrointest Liver Physiol 2012;303:G775–785. 22837345 10.1152/ajpgi.00155.2012

[4] Lovell RM Ford AC Global prevalence of and risk factors for irritable bowel syndrome: a meta-analysis. Clin Gastroenterol Hepatol 2012;10:712–21. e4. 22426087 10.1016/j.cgh.2012.02.029

[5] Gwee KA Gonlachanvit S Ghoshal UC Sencond Asian consensus on irritable bowel syndrome. J Neurogastroenterol Motil 2019;25:343–62. 31327218 10.5056/jnm19041 PMC6657923

[6] Pan G Lu S Ke M An epidemiologic study of irritable bowel syndrome in Beijing—a stratified randomized study by clustering sampling. Zhonghua Liu Xing Bing Xue Za Zhi 2000;21:26–9. 11860753

[7] Camilleri M Management options for irritable bowel syndrome. Mayo Clin Proc 2018;93:1858–72. 30522596 10.1016/j.mayocp.2018.04.032 PMC6314474

[8] Cangemi DJ Lacy BE Management of irritable bowel syndrome with diarrhea: a review of nonpharmacological and pharmacological interventions. Therap Adv Gastroenterol 2019;12:1756284819878950. 10.1177/1756284819878950 PMC6778998 31632456

[9] Song J Lei X Jiao W Effect of Qiangji Jianli decoction on mitochondrial respiratory chain activity and expression of mitochondrial fusion and fission proteins in myasthenia gravis rats. Sci Rep 2018;8:8623. 29872094 10.1038/s41598-018-26918-z PMC5988663

[10] Huang HP Pan H Wang HF Warming yang and invigorating qi acupuncture alters acetylcholine receptor expression in the neuromuscu lar junction of rats with experimental autoimmune myasthenia gravis. Neural Regen Res 2016;11:465–8. 27127487 10.4103/1673-5374.179060 PMC4829013

[11] Sun J Wu X Meng Y Electro-acupuncture decreases 5-HT, CGRP and increases NPY in the brain-gut axis in two rat models of Diarrhea-predominant irritable bowel syndrome(D-IBS). BMC Complement Altern Med 2015;29:340. 10.1186/s12906-015-0863-5 PMC4589130 26419631

[12] Dai Y-K Li D-Y Zhang Y-Z Efficacy and safety of modified Tongxie Yaofang in diarrhea-predominant irritable bowel syndrome management: a meta-analysis of randomized, positive medicine-controlled trials. PLoS One 2018;13:e0192319. 29408906 10.1371/journal.pone.0192319 PMC5800650

[13] Chinese Society of Digestive Diseases and Chinese Association of Chinese Medicine Consensus on the diagnosis and treatment of functional dyspepsia irritable bowel syndrome by Traditional Chinese Medicine. China J Trad Chin Med Pharm 2010;25:1062–5. 17.

[14] Leung WK Wu JC Liang SM Treatment of diarrhea-predominant irritable bowel syndrome with traditional Chinese herbal medicine: a randomized placebo-controlled trial. Am J Gastroenterol 2006;101:1574–80. 16863563 10.1111/j.1572-0241.2006.00576.x

[15] Sung JJ Leung WK Ching JY Agreements among traditional Chinese medicine practitioners in the diagnosis and treatment of irritable bowel syndrome. Aliment Pharmacol Ther 2004;20:1205–10. 15569124 10.1111/j.1365-2036.2004.02242.x

[16] Li Q Yang GY Liu JP Syndrome differentiation in chinese herbal medicine for irritable bowel syndrome:a literature review of randomized trials. Evid Based Complement Alternat Med 2013;2013:232147. 23554827 10.1155/2013/232147 PMC3608279

[17] Moher D Shamseer L Clarke M Preferred reporting items for systematic review and meta-analysis protocols (PRISMA-P) 2015 statement. Syst Rev 2015;4:1. 25554246 10.1186/2046-4053-4-1 PMC4320440

[18] Higgins JPT. Green S. Cochrane Handbook for Systematic Reviews of Interventions Version 5.1. 0 [updated March 2011]. Available at: http://www.cochrane-handbook.Org. Accessed June 1, 2020.

[19] Schmidt L Shokraneh F Steinhausen K Introducing RAPTOR: RevMan parsing tool for reviewers. Syst Rev 2019;8:151. 31242929 10.1186/s13643-019-1070-0 PMC6595567

[20] Ades AE Sculpher M Sutton A Bayesian methods for evidence synthesis in cost-effectiveness analysis. Pharmacoeconomics 2006;24:1–9. 10.2165/00019053-200624010-00001 16445299

[21] Salanti G Ades AE Ioannidis JP Graphical methods and numerical summaries for presenting results from multiple-treatment meta-analysis:an overview and tutorial. J Clin Epidemiol 2011;64:163–71. 20688472 10.1016/j.jclinepi.2010.03.016

[22] Song F Clark A Bachmann MO Simulation evaluation of statistical properties of methods for indirect and mixed treatment comparisons. BMC Med Res Methodol 2012;12:138. 22970794 10.1186/1471-2288-12-138 PMC3524036

[23] Sturtz S Bender R Unsolved issues of mixed treatment comparison metaanalysis: network size and inconsistency. Res Synth Methods 2012;3:300–11. 26053423 10.1002/jrsm.1057

[24] Sutton AJ Duval SJ Tweedie RL Empirical assessment of effect of publication bias on meta-analyses. BMJ 2000;320:1574–7. 10845965 10.1136/bmj.320.7249.1574 PMC27401

[25] Jing Yan Zhi-wei Miao Jun Lu Acupuncture plus Chinese herbal medicine for irritable bowel syndrome with diarrhea: a systematic review and meta-analysis. Evid Based Complement Alternat Med 2019;2019:7680963. 31110553 10.1155/2019/7680963 PMC6487118

[26] Manheimer E Wieland LS Cheng K Acupuncture for irritable bowel syndrome: systematic review and meta-analysis. Am J Gastroenterol 2012;107:835–47. 22488079 10.1038/ajg.2012.66 PMC3671917

[27] Xiao Y Liu Y Huang S The efficacy of Shugan Jianpi Zhixie therapy for diarrhea-predominant irritable bowel syndrome: a meta-analysis of randomized, double-blind, placebo controlled trials. PloS One 2015;10:e0122397. 25853241 10.1371/journal.pone.0122397 PMC4390216

